# The Study of Microbial Physiology Under Microoxic Conditions Is Critical but Neglected

**DOI:** 10.1111/1758-2229.70108

**Published:** 2025-06-16

**Authors:** Om Prakash, Ashvini Chauhan, Stefan J. Green

**Affiliations:** ^1^ Symbiosis Centre for Climate Change and Sustainability (SCCCS) Symbiosis International (Deemed University) Pune India; ^2^ Environmental Biotechnology Lab, School of the Environment Florida A&M University Tallahassee FL United States; ^3^ Department of Internal Medicine, Division of Infectious Diseases Rush University Medical Center Chicago Illinois USA

**Keywords:** bacteria, environmental genomics, environmental signal/stress responses, evolution/evolutionary processes/gene transfer/mutation, growth and survival, microbial ecology

## Abstract

During the early evolution of life on Earth, the environment was largely free of molecular oxygen, and only anaerobic life existed. With the subsequent oxidation of oceans and the atmosphere, a wide range of environmental niches, ranging from anoxic to microoxic/hypoxic and oxic, developed. Despite this broad range of natural environments, microbiology as a field has focused on the physiology, metabolism, and genetics of aerobic microorganisms, with less attention paid to anaerobes and much less attention paid to microaerophiles. The disparity in studies between aerobic and anaerobic conditions is rampant in host‐associated systems, particularly in human health, and studies of microorganisms in intermediate oxygen conditions between fully aerobic and fully anoxic conditions are exceedingly rare. Studies on the physiological behaviour, metabolism, growth response, and drug susceptibility patterns of commensal and pathogenic organisms are almost totally neglected in microoxic conditions. Furthermore, microorganisms from microaerobic and microoxic ecosystems have been less robustly explored in terms of physiology, growth, and metabolism. In this work, we highlight the importance of understanding the physiological and metabolic behaviours of microorganisms under hypoxic or microoxic conditions.

## Graphical abstract

Microbial physiology is mainly studied under aerobic or anaerobic/anoxic conditions, neglecting microbial behaviours in diverse microoxic conditions. The current article revisits the definition of microoxia in light of recent developments in oxygen‐sensing technology and emphasises the need to understand microbial physiology and growth behaviours of microbes in microaerobic/microoxic conditions with implications for environmental, climate and clinical studies.

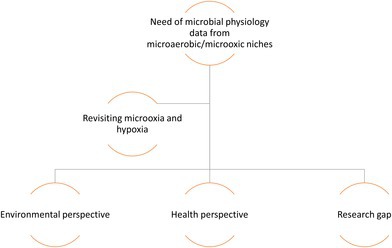

Levels of oxygen in Earth's atmosphere and oceans were exceedingly low until the ‘Great Oxidation Event’ around 2.4 to 2.3 Ga (Pufahl and Hiatt 2012). Although obligate anaerobes were likely the first kinds of organisms on Earth, the Last Universal Common Ancestor (LUCA) appears to have had exposure to low levels of molecular oxygen and hydrogen peroxide (Ślesak et al. 2012). Eventually, with full oxidation of oceans and the atmosphere, a broad range of ecosystems developed ranging from fully aerobic to microoxic/hypoxic to anoxic. Microorganisms evolved to colonise these niches and have evolved phenotypic responses to transient shifts in oxygen levels experienced in many ecosystems (Miralles‐Wilhelm and Gelhar 2000; Vanrolleghem et al. 2004). Despite this ecosystem diversity, cultivation microbiologists have been more inclined towards the study of aerobic microorganisms, albeit with substantial efforts made in the study of critical anaerobic processes such as methanogenesis, denitrification, sulphate‐reduction, and fermentation. Thus, while the focus of cultivation microbiology has been given to aerobic or anaerobic microbial growth, the cultivation of microorganisms that survive under varying oxygen concentrations between aerobic and anaerobic has been rather limited. This neglect of the study of microbial survival, tolerance, and physiology at intermediate oxygen concentrations is likely due to a number of factors, including (a) the lack/cost of sophisticated instrumentation for maintaining incubations at oxygen levels between full aerobic conditions to anaerobic conditions; (b) the complexity and time‐consuming nature of anaerobic/microaerophilic growth experiments; and (c) a lack of awareness of the importance of characterising microbial growth under intermediate oxygen conditions. Consequently, data about physiology, functionality, and behaviour of microorganisms at different oxygen concentrations is generally poor, even for microorganisms that have been submitted to culture collections.

According to historical definitions, obligate anaerobes are microbes that cannot survive in the presence of molecular oxygen (Table 1). These organisms were thought to lack defence mechanisms to protect themselves from the lethal effects of reactive oxygen species (ROS) generated during oxygen metabolism. However, recent studies have proved that similar to aerobic microbes, many anaerobes possess mechanisms to cope with the lethal effects of oxygen (Lu and Imlay 2021). Ecological studies have also identified ‘obligate anaerobes’ in areas with available oxygen, such as sulphate‐reducing bacteria in the oxygen chemocline of microbial mats (Minz et al. 1999), or have demonstrated capabilities for oxygen respiration (Cypionka 2000). Thus, it is likely that many ‘obligate anaerobes’ available in culture collections need to be reinvestigated for their oxygen tolerance abilities and survival potential in terms of extent and duration of exposure to varying oxygen concentrations. We recommend that the definition of ‘obligate anaerobes’ be redefined and that classifications of organisms based on their oxygen relationship be expanded.

**TABLE 1:** Definition of different oxygen conditions based on the presence and absence of oxygen or other terminal electron acceptors and oxygen concentrations.

Common terminology	Brief definition
Anaerobic	Biological systems, organisms or processes that do not require oxygen and operate using anaerobic respiration or fermentation.
Microaerobic	Biological systems, organisms or processes that need or operate at lower oxygen (generally 1%–10%) concentrations than atmospheric oxygen.
Nanaerobic	Biological systems, organisms or processes that need or operate at nanomolar levels of oxygen (< 1 μmol L^−1^).
Hyperaerobic	Oxygen levels exceeding saturation or atmospheric oxygen concentrations. Generally used for biological systems.
Dysaerobic	Refers to low oxygen levels of 0.1–2.0 mg/L. Term is generally used for biological systems.
Anoxic	Geological regimes/environmental ecosystems that lack oxygen as a terminal electron acceptor for aerobic respiration but do contain other common alternative electron acceptors for anaerobic respiration like NO_2_, SO_4_, and CO_2_.
Microoxic/hypoxic	Geological regimes/environmental ecosystems with oxygen concentrations below that of atmospheric oxygen and that also contain alternative electron acceptors like NO_2_, SO_4_, and CO_2_.
Nanoxic	Geochemical regimes or environmental ecosystems that contain nanomolar levels of oxygen (< 1 μmol L^−1^) along with alternative electron acceptors like NO_2_, SO_4_, and CO_2_.
Hyperoxic	Oxygen levels exceeding saturation levels or normal atmospheric oxygen concentration. Generally used for geological or environmental systems.
Dysoxic	Refers to low oxygen levels of 0.1–2.0 mg/L. Term is generally used for geological or environmental regimes.

## 1 Revisiting Microoxia and Hypoxia

The exact definition of the terms ‘microoxia’ or ‘hypoxia’ and its correlation with available oxygen levels is lacking in microbiology, geology and other related literature. The concept of hypoxia is broad, as any environment or niche with 0%–21% oxygen (below environmental oxygen level) can be defined as micro‐oxic/hypoxic, and any microorganisms that can grow with less oxygen than atmospheric oxygen are classified as microaerophilic (Table 1). Conversely, organisms that do not grow in the presence of or tolerate oxygen are called anaerobes. Berg et al. (2022) have compiled a table to define more narrowly oxygen terms, including aerobic, hyperaerobic/dysaerobic, microaerobic, subaerobic, nanoaerobic, and more. Furthermore, the development of low oxygen detection technology has revealed that ecological zones previously considered anoxic are more accurately described as microaerobic/microoxic or nanoaerobic/nanoxic, and that microorganisms previously considered to be obligate anaerobes should be considered as microoxic or nanaerobic. Specifically, iodometric titration‐based oxygen measurements are capable of measuring oxygen at concentrations below 2 μmol/kg, while other oxygen sensors have even lower detection limits (Berg et al. 2022). Commercially available oxygen detection systems have a wide range of limits of detection, including electrochemical dissolved oxygen sensors, electrochemical laser induced graphene‐based oxygen sensor (2.4 μM DO), optical dissolved oxygen sensors (0.1–0.2 mg/L), polarographic dissolved oxygen sensors (40 ppb to 40.00 ppm), solid‐state dissolved oxygen sensors (0.01 mg/L), Switch‐able Trace Oxygen (STOX) sensors (1–10 nM), and optical oxygen sensors (5 ppb). Using STOX and luminescence‐based sensors, a lower detection limit of 0.5–10 nmol/L oxygen has been obtained (Lehner et al. 2015; Revsbech et al. 2009). Additional devices have been developed for real‐time assessment of oxygen in tissues (Rivera et al. 2019), including polymer‐based sensors (Lin et al. 2024) which can have broad detection ranges from 5 ppm to 90% (Wu et al. 2023).

**FIGURE 1:**
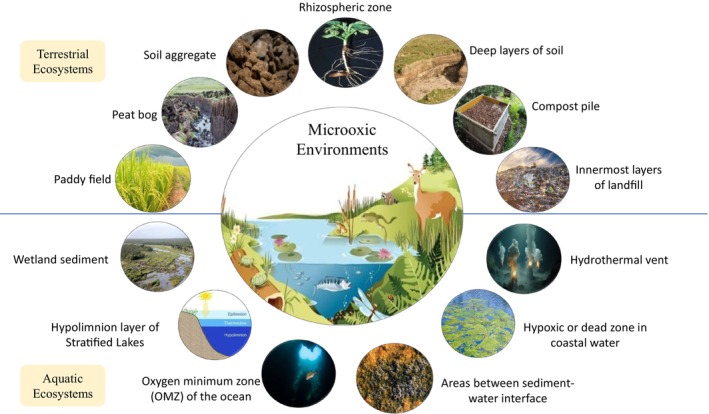
A pictorial representation of different microoxic niches in terrestrial and aquatic ecosystems.

Many microbial ecosystems, including soil, water, sediment, sludge, and animal and plant tissues, have oxygen availability below full oxygenation but above anoxia and include oxygen chemoclines (Tables 2 and 3). Based on prior studies of global oxygen availability, the most common microoxic environments found on Earth's surface include marine oxygen minimum zones (OMZs), stratified lakes, wetland soils and sediments, agricultural areas including leguminous root nodules and rice paddies, sediments, wastewater, anoxic micro‐niches within marine snow particles, and the gastrointestinal tracts of humans and other animals (Figure 1). Even “anaerobic” habitats, such as the mammalian gastrointestinal tract, saturated sediments, and hydrothermal vents, are not completely anaerobic, especially across localised micro‐gradients (Lu and Imlay 2021). These environments can experience episodic oxygenation, and organisms within these environments experience and survive intermittent oxygen exposure. Data from microoxic zones has indicated that the capacity to utilise and respire nanomolar oxygen is widespread among microbes which were previously considered anoxic (Kalvelage et al. 2015). In a survey of literature over decades, a wide range of tolerated oxygen concentrations in microaerophiles and ‘obligate anaerobes’ has been observed, with some organisms tolerating fully aerobic conditions (Table 2). This is consistent with the concept that most ‘obligate anaerobes’ need some traces of oxygen for growth and survival, and that some ‘obligate anaerobes’ can grow in the presence of oxygen. Similarly, when surveying measured oxygen levels in environmental and host‐associated locations, a broad range of hypoxic conditions are found (Table 3), while nearly all environments can experience conditions in the microaerophilic range intermittently. The prior categorization of many ‘obligate anaerobes’ is no longer consistent with decades of observation and reporting, and microbiologists need to carefully assess both oxygen measurements using improved sensing devices and address the need for reclassification of organisms and environments according to oxygen tolerance and presence, respectively.

**TABLE 2:** Oxygen requirements for select microbial taxa.

No.	Name of the microorganisms	Minimum oxygen requirement for growth (%)	References
Microaerophilic microorganisms			
1.	*Aspergillus fumigatus*	0.5–2.5	(Hall and Denning 1994)
2.	*Azospirillum brasilense*	0.4	(Bible et al. 2015)
3.	*Burkholderia cenocepacia H111*	0.5–5.0	(Pessi et al. 2013)
4.	*Campylobacter jejuni*	2.0–10	(Kaakoush et al. 2007), (Rodrigues et al. 2015)
5.	*Vibrio succinogenes*	2.0	(Wolin et al. 1961)
6.	*Candidatus Ovobacter propellens*	0.5	(Fenchel and Thar 2004)
7.	*Galenea microaerophila* gen. nov., sp. nov.	5.0	(Giovannelli et al. 2012)
8.	*Helicobacter pylori*	5.0–15.0	(Perez‐Perez et al. 2016)
9.	*Herbaspirillum seropedicae SmR1 Fnr*	< 0.5	(Batista et al. 2013)
10.	*Lactobacillus sake*	20.0	(Amanatidou et al. 2001)
11.	*Magnetospirillum gryphiswaldense MSR‐1*	0.5–5.0	(Zhuang et al. 2017)
12.	*Mycobacterium genavense*	2.5–5.0	(Realini et al. 1998)
13.	*Neisseria gonorrhoeae*	0.05–0.15	(Kellogg et al. 1983)
14.	*Paracoccidioides* (*Yeast*)	1.0	(Lima et al. 2015)
15.	*Plasmodium falciparum*	0.5–5.0	(Torrentino‐Madamet et al. 2011)
16.	*Porphyromonas gingivalis*	6.0	(Lewis et al. 2009)
17.	*Pseudomonas aeruginosa*	0.4–20.0	(Alvarez‐Ortega and Harwood 2007)
18.	*Legionella* spp.	0.00002–0.0015	(Nguyen et al. 1991)
19.	*Rhizobium leguminosarum bv. viciae 3841*	1.0–21.0	(Wheatley et al. 2017)
20.	*Salmonella enterica* serovar *typhimurium*	20	(Jennewein et al. 2015)
21.	*Spirillum* sp. Str. *5175*	2.0	(Schumacher et al. 1992)
22.	*Spirillum volutans*	< 12.0	(Padgett et al. 1982)
23.	*Staphylococcus epidermidis*	0.0–5.0	(Uribe‐Alvarez et al. 2016)
24.	*Treponema pallidum*	0–3.0	(Fitzgerald 1981)
25.	*Wolinella succinogenes*	2.0	(Baar et al. 2003)
Obligate anaerobic microorganisms			
26.	*Desulfovibrio vulgaris*	0.04–0	(Johnson et al. 1997)
27.	*Pyrococcus furiosus*	8.0	(Thorgersen et al. 2012)
28.	*Geobacter sulfurreducens*	5.0–10.0	(Lin et al. 2004)
29.	*Bacteroides caccae*	0.03	(Baughn and Malamy 2004)
30.	*Bacteroides distasonis*	0.03	(Baughn and Malamy 2004)
31.	*Bacteroides ovatus*	0.03	(Baughn and Malamy 2004)
32.	*Bacteroides thetaiotaomicron*	0.03	(Baughn and Malamy 2004)
33.	*Bacteroides uniformis*	0.03	(Baughn and Malamy 2004)
34.	*Bacteroides vulgatus*	0.03	(Baughn and Malamy 2004)
35.	*Bacteroides fragilis*	0.1–0.2	(Baughn and Malamy 2004)
36.	*Bacteroides oralis*	< 0.4	(Tally et al. 1975)
37.	*Bacteroides melaninogenicus*	< 2.5	(Tally et al. 1975)
38.	*Faecalibacterium prausnitzii*	20	(Khan et al. 2012)
39.	*Clostridium sordellii*	7.5	(Tally et al. 1975), (Rolfe et al. 1977)
40.	*Clostridium putrificum*	10.0	(Tally et al. 1975), (Rolfe et al. 1977)
41.	*Clostridium perfringens*	6–8	(Tally et al. 1975), (Rolfe et al. 1978)
42.	*Peptostreptococcus elsdenii*	> 2.5	(Tally et al. 1975)

*Note:* Equivalent oxygen concentrations in different units (%, ppm and mg/L): 1% = 10,000 ppm = 9988.6 mg/L.

**TABLE 3:** Different habitats and their oxygen concentrations.

Ecosystems	Oxygen conc. (%)	Ecosystems	Oxygen conc. (%)
Soil	0.04–21.0	Marine water at temp. 15.5°C	0.0009671
Normal water at standard temperature (25°C)	0.00065–0.0008	Marine water at temp. 21.1°C	0.0008689
Marine water (35 ppt salt) at standard temperature (25°C)	0.0007007–0.0008009	Activated sludge	0.00005–0.00030
Altitudes (data from (Peacock 1998))			
0.0 m	20.9	4572.0 m	11.8
304.8 m	20.1	4876.8 m	11.4
609.6 m	19.4	5181.6 m	11.0
914.4 m	18.6	5486.4 m	10.5
1219.2 m	17.9	5791.2 m	10.1
1524.0 m	17.3	6096.0 m	9.7
1828.8 m	16.6	6400.8 m	9.4
2133.6 m	16.0	6705.6 m	9.0
2438.4 m	15.4	7010.4 m	8.7
2743.2 m	14.8	7315.2 m	8.4
3048 m	14.3	7620.0 m	8.1
3352.8 m	13.7	7928.8 m	7.8
3657.6 m	13.2	8229.6 m	7.5
3962.4 m	12.7	8534.4 m	7.2
4267.2 m	12.3	8839.2 m	6.9
Gastrointestinal tract (data from (Singhal and Shah 2020)			
Colonic muscle wall	7.0–10.0	Lumen of ascending colon	2.0
Small intestinal wall	8.0	Sigmoid colon	0.4
Small intestinal lumen	2.0	Villus tip	3.0
Vascularized submucosa	6.0		
Mammalian body niches (data from (Gan and Ooi 2020) and references therein)			
Trachea	19.7	Kidney (rat)	5.9–6.6
Arterial blood	13.2	Placenta	7.4 ± 0.4
Venous blood	5.3	Umbilical cord	2.7–3.9
Brain	4.4	Umbilical artery	1.3–1.9
Normal lung	5.6	Bone marrow	7.22 ± 0.1
Lung tumour	0.1–6.1	Ovaries	11.6
Skin (epidermis)	1.1 ± 0.42	Spleen	10.0 ± 2.4
Skin (dermal papillae)	3.15 ± 0.8	Lymphoid organs	0.5–4.5
Liver	7.5 ± 0.7	Skeletal muscle	3.3 ± 0.58
Kidney (human)	6.8 ± 0.8	Adipose tissue	4.7–8.9

*Note:* Equivalent oxygen concentrations in different units (%, ppm and mg/L): 1% = 10,000 ppm = 9988.6 mg/L.

That so many ecological niches have oxygen concentrations below that of saturation or atmospheric concentrations indicates the significance of the study of microbial physiology and functionality under microoxic conditions. We note, however, most studies of microbial physiology, including growth response, generation time, biochemical traits, and gene expression, are conducted on cultures grown on agar plates or liquid media under atmospheric oxygen concentrations or in the complete absence of oxygen in anaerobic chambers or sealed microcosms. Due to the difficulty of sustaining intermediate oxygen concentrations in the presence of microbial metabolism, studies under reduced oxygen concentrations are rare, leading to a disconnect between laboratory physiological characterisation and in situ activity. Furthermore, Berg et al. (2022) indicated that in the future, many of Earth's ecosystems will shift towards anoxic and microoxic conditions due to intense anthropogenic interventions such as the discharge of wastewater or agricultural runoff. Therefore, studying the range and optima of oxygen for the growth of microaerophiles and obligate aerobes and oxygen lethality in terms of duration and concentration for ‘obligate anaerobes’ is essential. A better understanding of microbial metabolism under reduced oxygen conditions, including microoxic and anoxic environments, will be essential to address global issues related to climate change, stress tolerance, pollution remediation, disease progression, and sustainable development.

## 2 Environmental Implications of Microoxia

Research has demonstrated that many ecosystems contain microoxic or anoxic zones, with the exception of the lowermost layer of Earth's atmosphere, that is, the troposphere (Table 3). However, the partial pressure of oxygen decreases with increasing altitude and creates conditions of hypoxia or anoxia (Table 2), and this can affect the viability of airborne organisms (Mohr 2007). In contrast, diffusion of oxygen in water and loss of aerobic photosynthesis in surface waters leads to conditions of hypoxia with increasing depth in water columns (Hietanen et al. 2012; Jane et al. 2023). In addition, deposition of organic matter, high respiration rates, algal blooms, organic pollutants, and low rates of photosynthesis can exacerbate hypoxic conditions in aquatic ecosystems. Such hypoxia can kill large eukaryotic organisms, including fish, worms, and molluscs (Diaz 2001; Rabalais et al. 2002), though less attention has been given to negative impacts on micro‐eukaryotic life such as rotifers, ciliates, and protozoans (Cai et al. 2018).

Similarly, prominent terrestrial ecosystems that are characterised by microoxic or anoxic conditions include paddy fields, peat bogs, soil aggregates, some rhizosphere zones, deep layers of soil, compost piles, and lower and innermost layers of landfills (Berg et al. 2022; Bertagnolli and Stewart 2018; Diaz 2001; Trojan et al. 2021). Without intervention, deeper layers of soil are expected to be microoxic due to the reduced rate of diffusion of oxygen in the lower soil strata. In plant root rhizospheres, the level of oxygen generally exceeds that of bulk soil due to oxygenation through the root system (Colmer 2003; Revsbech et al. 2009), and conduits created by the root also facilitate more oxygen diffusion into the root zones (rhizosphere) relative to bulk soil. However, intense microbial activity in the rhizosphere can rapidly consume oxygen, leading to transient and localised reduced oxygen levels (Garcia Arredondo et al. 2024; Keiluweit et al. 2018). Many factors can contribute to soil oxygen depletion, including fast rates of microbially‐mediated organic matter degradation, excess accumulation of organic load, soil texture, tillage practices, overgrazing, root respiration, waterlogging, plant species, and more (Siedt et al. 2023, Lussich et al. 2024). Microoxic conditions of soil impact microbial community structures, nutrient availability, biogeochemical cycling, soil health, and crop productivity. Oxygen limitation in soils leads to slower degradation of organic matter and can promote anaerobic metabolism including fermentation, methanogenesis (Angel et al. 2012), sulphate‐reduction, and denitrification (Butterbach‐Bahl and Dannenmann 2011). Although anoxic conditions limit carbon oxidation in peatlands (Liu et al. 2023) and are essential for fixation of nitrogen through nitrogenase activity in leguminous plant root nodules (Rutten and Poole 2019), anaerobic microbial metabolism can reduce soil fertility and lead to the release of greenhouse gases like CO_2_, CH_4_, and N_2_O (Rohe et al. 2021). Water‐logged soils such as paddy fields are well known for methanogenesis and methanotrophy (Conrad 2020). In general, microoxia can hamper microbially‐driven biogeochemical cycling of materials in agricultural soil, lead to microbial community shifts towards facultative anaerobes, trigger biodiversity loss, promote nitrate depletion, lead to excess release of greenhouse gases, and impact crop health, agricultural productivity, and climate change (Deng et al. 2022, Sun et al. 2023, Lussich et al. 2024). Thus, improved understanding of soil oxygen levels and related microbial functionality is imperative for sustainable soil management, agriculture, and climate risk mitigation.

Similar to the deeper layers of soil, water columns of aquatic ecosystems often contain lower oxygen relative to terrestrial and atmospheric environments. Some of the most prominent microoxic niches of aquatic ecosystems include the oxygen minimum zone (OMZ) of the ocean, hydrothermal vents, hypoxic or dead zones in coastal waters, the sediment–water interface, hypolimnion or deeper colder layers of stratified lakes and wetland sediments, among others. These environments can be affected through local and global processes (Zhang et al. 2010), including excess nutrient accumulation/organic matter, thermal stratification, salinity, organic matter degradation in sediment, hydrology like stagnant water, urbanisation and agricultural practices, all of which can promote microoxia or anoxia in aquatic habitats. In these environments, a variety of critical microorganisms facilitate carbon and nutrient cycling, including denitrifiers, sulphate‐reducers, methanogens, anaerobic ammonium oxidation (ANNAMOX) microorganisms (Kuenen 2008), and anaerobic methanotrophic archaea (ANME; Evans et al. 2019).

Aquatic sediments too can be dominated by hypoxic or anoxic conditions (Middelburg and Levin 2009). For example, mangroves are aquatic ecosystems characterised by salinity, high organic matter degradation, vegetation with a dense root system, and continuous water saturation (Palit et al. 2022). These features of mangroves prevent normal oxygen diffusion, and its rapid consumption due to intense microbial processes leads to microoxia and anoxia in sediment and water columns (Alongi 2005). Microoxic conditions of mangroves similarly promote anaerobic microbiological processes like denitrification, sulphate reduction, and methanogenesis, which can release toxic and greenhouse gases into the environment, but can also serve as carbon sinks (Cameron et al. 2019). Non‐saline aquatic wetlands also play important roles in water purification and are one of the biggest sinks of environmental carbon (Hopkinson et al. 2012). While mangrove and freshwater wetlands play important roles in environmental carbon sequestration, they can also contribute to global processes through the release of greenhouse gases due to intense microbial activities in their microoxic and anoxic pockets (Alongi 2012) leading to further needs for evaluation of microbial physiology under intermediate and transient oxygen conditions.

## 3 Role of Microoxia in Disease Pathogenesis

Anaerobic infections and conditional or transient drug resistance are emerging areas in clinical microbiology (Gajdács and Urbán 2020). Abscesses of the lungs, brain, and gastrointestinal tract (GIT), deep wounds, diabetic foot ulcers, and vaginal tracts can experience hypoxic to anoxic conditions (Table 3). For example, invasive or systemic fungal infections of deep internal organs like the kidney, brain, heart, and GIT have a high rate of morbidity and mortality in immunocompromised patients (Chmel et al. 1993; Garnacho‐Montero et al. 2024). Initially, these infections are hypoxic in nature, but the rapid growth of aerobes and facultative anaerobes consumes available oxygen and makes the infection site anaerobic and amenable to the growth of anaerobic pathogens (André et al. 2022). Hypoxic or anoxic conditions of infection sites can create a stress response, which induces a transient change in the morphology, physiology, and growth rate of pathogens, leading to variable drug susceptibility (Yadav et al. 2023; Kovale et al. 2021; Gupta et al. 2016; Wallace et al. 2016; Ernst and Tielker 2009). Hypoxia can benefit pathogens by the reduction of reactive oxygen species (ROS) and neutrophil extracellular traps (NETs; André et al. 2022). Strains of the facultative anaerobic fungus *Scedosporium apiospermum*, which are frequently reported in cases of invasive fungal infections from immunocompromised patients, showed variable drug responses in aerobic versus anaerobic conditions, and tested strains showed two‐ to four‐fold higher sensitivity under anaerobic conditions relative to aerobic conditions (Yadav et al. 2023). In a study of the sensitivity of a range of gut‐derived facultative anaerobic bacteria to antibiotics under aerobic and anaerobic conditions, resistance varied widely by organism, antibiotic type, and the presence or absence of oxygen (Kovale et al. 2021). Similarly, (Gupta et al. 2016) observed strain‐specific effects of different oxygen levels on antibiotic susceptibility of *Staphylococcus aureus, Pseudomonas aeruginosa*, and *Klebsiella pneumoniae* isolates. Although most antibiotic testing and dosage optimisation is performed in the presence of oxygen (Sønderholm et al. 2017), some microbial strains have shown drug sensitivity under aerobic conditions while completely resistant under anaerobic conditions (e.g., faeces‐derived *Kocuria indica* strain LY94 with Ceftriaxone; Kovale et al. 2021). Furthermore, Grahl et al. (2011) observed the development of hypoxia in a pulmonary invasive fungal infection and concluded that fungal production of ethanol by fermentation under microaerobic lung conditions contributed to fungal pathogenesis (Xiu et al. 2022). Conversely, induced hypoxic microenvironments have been used to treat bacterial biofilm infections of *S. aureus* , as the metabolism of methicillin‐resistant *S. aureus* under anaerobic conditions led to increased sensitivity to metronidazole.

The field of conditional or transient drug response of microorganisms to hypoxia or anoxia is still in its infancy, and data are available only for a limited number of microorganisms. In addition, studies as to how hypoxia or anoxia changes the inflammatory response, outcome of antibiotic therapy, and pathogenesis are essential and will have a substantial impact on clinical practices. Experiments with a broader array of pathogenic strains, multiple classes of antibiotics, and across different oxygen conditions are needed to improve treatment regimens.

## 4 Perspective

The distribution of hypoxic and microoxic conditions has expanded due to recent improvements in oxygen‐sensing technology. The existing definitions of hypoxic or anoxic microorganisms are vague or unclear, and a more robust understanding of the activity of microorganisms in intermediate oxygen conditions has yet to be obtained. Indeed, some current ‘obligate anaerobes’ tolerate oxygen or use some oxygen for energy generation. Most characterisation of microorganisms, however, is performed either under fully aerobic or anaerobic conditions, leaving substantial knowledge gaps under intermediate oxygen conditions. It is further necessary to improve naming conventions to characterise growth conditions of microaerophiles, and we recommend that new naming conventions should include well‐defined subcategories based on levels of oxygen tolerance (nanoaerophiles, microaerophiles, megaaerophiles, etc.). Cultivability of environmental and host‐associated microorganisms may further be improved using a range of oxygen conditions for enrichment and isolation. The role of hypoxia in human pathogenesis by bacterial and fungal pathogens needs further attention, and a better understanding of microbial physiological response to hypoxia and anoxia may lead to drug treatment regimens tailored to the pathogen and body site, identify causes for drug treatment failure, allow for novel treatments, and improve patient outcomes.

Overall, expanded knowledge of the growth and metabolic behaviours of microorganisms across a range of intermediate oxygen conditions will provide essential data regarding clinical, industrial, agricultural, and environmental applications. It will further assist in the collection, long‐term preservation, and revival of microorganisms in culture collections, and change sample collection, transport, and storage strategies. Clinically, a better understanding of the relationship between oxygen, biofilm formation, metabolism, and antibiotic resistance across the realm of environmental and pathogenic microorganisms is much needed. The use of improved instruments to measure low oxygen concentrations is further necessary to accurately assess the growth, survival, and physiology of microorganisms in response to low but available levels of oxygen.

## Author Contributions

**Om Prakash:** conceptualization, data curation, formal analysis, investigation, validation, writing – original draft. **Ashvini Chauhan:** visualization, writing – review and editing. **Stefan J. Green:** validation, visualization, writing – review and editing.

## Conflicts of Interest

The authors declare no conflicts of interest.

## Data Availability

Data sharing is not applicable to this article as no new data were created or analyzed in this study.
